# Dermatological implications of alignment-based de-hosting and bioinformatics pipelines on shotgun microbiome analysis

**DOI:** 10.1186/s12967-025-07246-z

**Published:** 2025-11-13

**Authors:** Daniela Orschanski, Leonardo Néstor Rubén Dandeu, Martín Nicolás Rivero, Vivian Labovsky, Elmer Andrés Fernández

**Affiliations:** 1 ScireLab - Fundación para el Progreso de la Medicina, Consejo Nacional de Investigaciones Científicas y Técnicas (CONICET), Córdoba, Argentina; 2 Facultad de Ciencias Exactas, Físicas y Naturales (FCEFyN), Universidad Nacional de Córdoba (UNC), Córdoba, Argentina; 3 Laboratorio de Inmunohematología, Instituto de Biología y Medicina Experimental (IBYME), Fundación IBYME, Consejo Nacional de Investigaciones Científicas y Técnicas (CONICET), Ciudad Autónoma de Buenos Aires, Buenos Aires, Argentina; 4 Instituto de Farmacología, UBA Buenos Aires, Buenos Aires, Argentina; 5 Universidad Argentina de la Empresa (UADE). Instituto de Tecnología (INTEC), Lima 717, Buenos Aires, Argentina

**Keywords:** Bioinformatics pipelines, Shotgun metagenomics, Taxonomic classification, Microbial profiling, De-host

## Abstract

**Background:**

The skin microbiome is a critical component of dermatological health, with its dysbiosis implicated in conditions ranging from atopic dermatitis to cancer. Shotgun metagenomics offers an unparalleled resolution for comprehensive taxonomic and functional profiling, yet its application in dermatology is hampered by the high proportion of host DNA and the lack of consensus on best-practice bioinformatic pipelines. While Illumina’s proprietary DRAGEN platform is widely used, its closed-source nature and cost limitations necessitate the validation of robust, open-source alternatives to democratize access and enable customization.

**Methods:**

This study evaluates the performance of Kraken-based open-source pipeline as a viable alternative to the DRAGEN platform as well as the effect of currently available alignment-based de-hosting methods—Bowtie2, BWA, and Rsubread—to remove human DNA, assuring the use of highly-curated human reference genome thus avoiding the limitations of potentially incomplete or contaminated k-mer-based databases. By using shotgun metagenomic data from 83 healthy individuals we systematically compared the impact of these de-hosting procedures prior to Kraken2/DRAGEN taxonomic classification and functional profiling using HUMAnN 3.0 to assess the influence of methodological choices on skin microbial community composition and metabolic pathway abundance interpretation.

**Results:**

Our analysis revealed marked discrepancies arising from the choice of de-hosting tool and taxonomic classifier, leading to substantial variability in microbial and functional profiles that could compromise clinical interpretation. Among the pipelines tested, Bowtie2 de-hosting combined with Kraken2 taxonomic classification and HUMAN functional profiling efficiently recovered well-established sex- and age-related bacterial associations in healthy skin that were missed by all other methods, including DRAGEN. This superior performance, together with its customizable features, underscores the value of this workflow for robust and clinically relevant dermatological metagenomic studies.

**Conclusions:**

Our findings underscore the decisive impact of bioinformatic pipeline selection on skin microbiome analysis and offer actionable guidance for reproducible and clinically meaningful research. We present a customizable workflow that enhances reproducibility and transparency while improving the translational value of metagenomic data. This approach strengthens the reliability of microbiome studies and supports the development of precision diagnostics and personalized therapeutic strategies in dermatology.

**Supplementary information:**

The online version contains supplementary material available at 10.1186/s12967-025-07246-z.

## Background

The skin microbiome plays a pivotal role in maintaining dermatological health, with dysbiosis linked to conditions such as atopic dermatitis, psoriasis, acne and cancer [1–3]. Its impact is mediated through mechanisms like the production of immunomodulatory metabolites [4], competitive inhibition of pathogens, modulation of inflammatory responses, and maintenance of the skin barrier [5].

Metagenomic analysis has emerged as a powerful tool to unravel microbial contributions to skin pathophysiology, providing insights into immune modulation, barrier function, and potential therapeutic targets [6]. While 16S rRNA sequencing has been widely used for taxonomic profiling [7], shotgun metagenomics provides superior resolution, enabling species- and strain-level identification of bacteria, viruses, and fungi, as well as their functional pathway analysis [8]. Despite its advantages, shotgun metagenomics remains underutilized in skin microbiome research compared to amplicon-based methods, mainly due to its higher costs and the substantial amount of human DNA, a critical challenge in skin metagenomics that requires computational removal necessary to focus on microbial sequences [9]

Several taxonomic classifiers (e.g. DRAGEN [10], Kraken2 [11] STAT [12], MetaPhlAn4 [13], etc) have been developed for analysing taxonomic abundance from the growing volume of sequenced data [14]. However, a consensus on best-practice pipelines for dermatological applications focused on shotgun metagenomics is still lacking, particularly in real-world scenarios where no gold standard is available. Currently, Illumina’s DRAGEN metagenomics analytical platform has become a widely adopted tool for taxonomic classification in clinical and commercial applications [15], largely due to its straightforward integration of both Illumina technologies: sequencer and BaseSpace® platform. Despite this effective integration and speed processing capabilities, it presents important limitations such as cloud dependency, proprietary built-in de-hosting and taxonomic database (both limiting pipeline customization) and high costs. Therefore accounting for reliable and customizable real open-source alternatives that overcome such limitations and democratize its access turns crucial in clinical dermatology. This implies to disentangle the two main processes: de-hosting and taxa classification. Motivated by the DRAGEN technical documentation mentioning that it relies on Kraken2 algorithms and taxonomic databases (TxDB), we wonder if the current Kraken 2 software may become such an open-source alternative for tax classification. In terms of the de-hosting procedure, it may rely on two main approaches, k-mer or alignment based. The former requires the use of an extensive and highly completed taxonomic reference TxDB holding a full repertoire of the host/human genome. However, current TxDB may hold human contaminated microbial genomes or it may be incomplete [16], a fact usually unknown for end users. On the other hand, alignment-based de-hosting relies on explicitly providing the host genome reference, allowing, in the human case, to provide a well known, trustable, highly curated, easily accessible and widely used resource.

Based on this and with the fact that there is no consensus on optimal bioinformatic analytical pipelines [17], the impact of alignment-based de-hosting methods is explored as a prior step for downstream taxonomic and functional analysis in skin metagenomics. Here, the evaluation of Kraken2 as an equivalent of DRAGEN Metagenomics Pipeline is explored by comparing both tools fed by alignment-based de-hosting methods (e.g., Bowtie2 [18], BWA [19], Rsubread [20], in-built DRAGEN) for taxonomic analysis. In addition, since metabolic information is crucial for dermatological treatment indications, de-hosting impact was also evaluated over the widely used HUMAnN 3.0 microbial pathways profiling tool [21].

In this study, shotgun metagenomic data from 83 healthy individuals were used to demonstrate that de-hosting and tax-classification methods significantly alter microbial community and metabolic pathway descriptions and that Kraken, appropriately attached to well chosen de-hosting procedures, is a highly competing alternative bioinformatic pipeline to DRAGEN metagenomic proprietary software. Our findings provide a framework tailored to specific research or clinical objectives, ensuring robust and reproducible interpretation of skin microbiome data. By uncovering key methodological biases, this work enables researchers and clinicians to optimize metagenomic workflows, ultimately enhancing precision in dermatological diagnostics and therapeutic strategies.

## Methods

### Study design and cohort

83 facial skin samples were collected from healthy individuals and sequenced using Illumina Nextera XT (Gencove), generating paired-end FASTQ files (2 × 150 bp). Metadata including sex and age range were available for all participants, with 45 females and 38 males. Participants were classified into three age groups: 18–35 years, 36–55 years, and over 55 years. All participants had no reported pathological conditions and presented with clinically normal skin. Inclusion criteria included healthy individuals with Fitzpatrick skin phototypes II to IV. Exclusion criteria were systemic antibiotic use within one month prior to sampling, topical skin treatments (e.g., anti-acne creams, lotions, or soaps) within two weeks before sampling, hormonal contraceptive use and obesity defined as a body mass index (BMI) above 30.

## Data processing

**Fig. 1:**
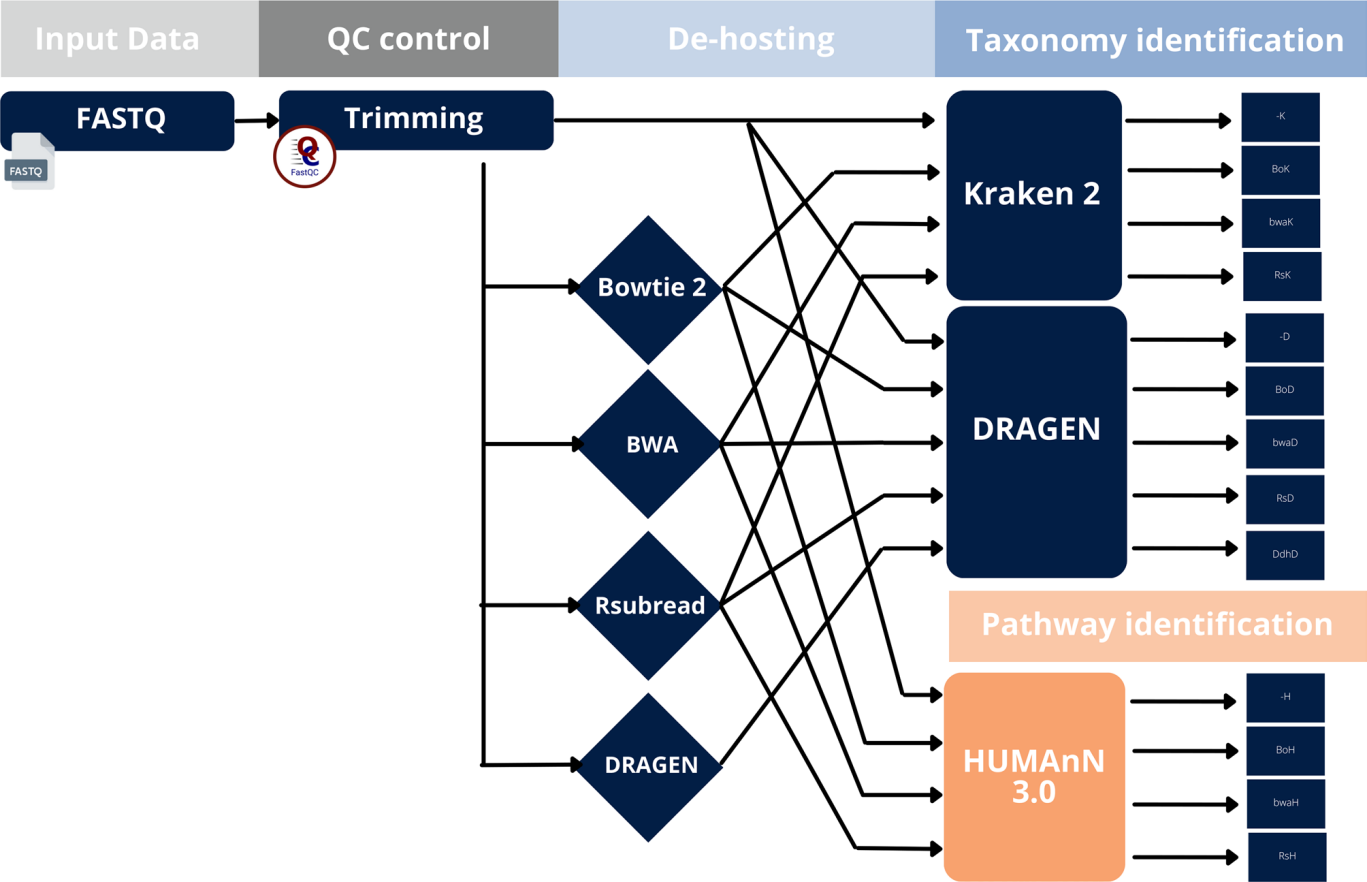
Bioinformatics workflow. Four pipelines for Kraken (-K, BoK, bwaK and RsK), five for DRAGEN (-D, BoD, bwaD, RsD and DdhD: de-hosted by proprietary DRAGEN de-hosting feature) and four for HUMAnN (-H, BoH, bwaH and RsH)

Each sample was processed through multiple pipelines to assess variability introduced by different bioinformatic methodologies. The proposed pipeline is presented in Fig. 1 and implies the following tasks:

- Quality Control (QC): sequence quality was assessed using FastQC v0.12.1 [22]. Reads with an average quality score < 20 were trimmed using TrimGalore [23].
- De-hosting: four alignment-based human DNA removal techniques were implemented in this study. BWA, Bowtie 2, Rsubread and DRAGEN’s proprietary de-hosting functionality (the latter only available for Illumina´s DRAGEN Metagenomic Pipeline). All tools were applied using default parameters (See: Supplementary Material - Code Reproducibility) to identify and remove reads mapped to the human reference genome (GRCh38, Ensembl Release 110) [24]. After quality filtering, raw FASTQ files either proceed directly to taxonomic or functional profiling tools or first go through a de-hosting step. For software versions see Table 1.
- Taxonomic identification: To assess downstream impact on taxonomic classification, we used two classifiers: the DRAGEN Metagenomics Pipeline and Kraken 2, which DRAGEN reports as the basis for its algorithms and database. In order to align with the available settings in the DRAGEN Metagenomics Pipeline interface, Kraken2 was fed with the same taxonomic database—Minikraken v2 (March 2020 download from [25]), a reduced and memory-efficient version of the full Kraken2 database. By means of this, a consistent reference and an equivalent comparison of taxonomic classification performance is ensured for both tools. This database includes bacterial, archaeal, viral, and human sequences. Kraken2 and DRAGENs softwares were used to process the output FASTQ files from the de-hosting and un-dehosted strategies, thus yielding four different pipelines for Kraken 2 (-K : un-dehosted), BoK : de-hosted by Bowtie 2, bwaK : de-hosted by BWA and RsK: de-hosted by Rsubread R package) and five for DRAGEN. (-D, BoD, bwaD, RsD and DdhD: de-hosted by proprietary DRAGEN de-hosting procedure).
- Pathway identification: For functional profiling, we used HUMAnN 3.0 to evaluate the impact of de-hosting on results directly relevant to dermatological treatment decisions. HUMAnN 3.0, the next iteration of the HUMAnN tool from the HMP Unified Metabolic Analysis Network. This tool takes the same input data as Kraken 2 or DRAGEN: FASTQ files that have either been de-hosted or simply quality filtered, resulting in five different pipelines for comparison (-H: un-de-hosted, DoH: de-hosted by Bowtie 2, bwaH: de-hosted by BWA and RsH: de-hosted by Rsubread). This software uses MetaCyc as a database [26], which contains pathways involved in both primary and secondary metabolism, as well as associated metabolites, reactions, enzymes, and genes.

**Table 1:** Comparison of usability and computational features characteristics across methodologies

	De-hosting Aligners			Taxonomy			Functional
	Bowtie 2	BWA	Rsubread	DRAGEN GUI	DRAGEN CLI	Kraken 2	HUMAnN 3.0
Version	2.4.5	0.7.17	2.8.2	3.5.12	1.6.1	2.1.5	3.0
Web access	x	x	x	✓	x	x	x
Internet connection needed	x	x	x	✓	✓	x	x
Native code	Perl and C++	C	R	FPGA + Software propietario		C++	Python
Bioinformatic skills	High	High	High	Low	High	High	High
External installation	✓	✓	x	x	✓	✓	✓
De-host Feature	-	-	-	✓	✓	x	x
Control over output format	✓	✓	✓	x	x	✓	x
Multiprocessing	✓	✓	✓	x	x	✓	✓
Index Reference	x	✓	✓	x	x	-	-
Runtime per sample (seconds)	57	61	37	*	*	2.5 (de-hosted) 4.5 (without de-hosting)	9.3 (de-hosted) 14.3 (none-dehosting)

* Utilizing a free DRAGEN version, its analysis is influenced by the server times and its data uploading limits

Supplementary Table S1 shows the thirteen methodologies generated by combining different alignment-based de-hosting methods with taxonomy identification tools and with the pathway identification tool. The resulting methodology includes the abbreviations of the tool names to provide a clear mapping of the different methodological combinations.

## Statistical analysis

Comparisons across multiple groups were performed using the Kruskal-Wallis test. Wilcoxon tests were used for pairwise comparisons: paired tests when comparing methods applied to the same samples, and unpaired tests for comparisons between different groups of samples. *P*-values were adjusted for multiple testing using the Benjamini-Hochberg (BH) method. Differences were considered statistically significant at *p* < 0.05.

Since no gold standard is available for skin metagenomics analysis, the true microbial composition is unknown. Therefore, in order to evaluate Illumina’s DRAGEN metagenomic performance, Bland-Altman plots were used to assess agreement between competing methods (DRAGEN vs Kraken based pipelines) in abundance estimations across different de-hosting procedures. The Bland-Altman plots provide a graphical representation of the magnitude and direction of differences between methods [27]. Then, with the aim to evaluate clinical impact of both analytical pipelines, i.e taxonomic classification and functional analysis, age- and sex-associated taxa and metabolic pathways were used and their associated patterns validated against well known publications references.

These approaches are aimed to identify open-source alternatives that preserve biologically relevant information and support reliable, transparent analysis in dermatological analysis.

All statistical analyses were performed in R (version 4.1.2) using the appropriate statistical packages.

## Results

### Comparison of usability

Each pipeline provides an avenue for analysis of shotgun metagenomic sequencing samples. However, there are major variations in the implementation of each one (Table 1). For taxonomic characterization two main tools are commonly used: the DRAGEN Metagenomic Pipeline and Kraken 2. DRAGEN offers two formats of its use: one consists of a web-based graphical user interface (GUI) and the other is based on command line interface (CLI) [28]. In the web format, the uploaded data automatically undergoes several analytical steps, leaving the user to generate abundance profiles, functional features, and visualizations. Meanwhile, BaseSpace Sequence Hub CLI supports programmatic access to BaseSpace Sequence Hub for automation. In both cases the user must first upload the FASTQ files in a specific format, run the process, and finally download the results. The user can enable the proprietary de-hosting procedure. DRAGEN does not require high computing resources, but does require a stable internet connection.

On the other hand, Kraken 2 is a command-line-based algorithm in which the user runs a set of sequential scripts to achieve classification in a custom or default database. Kraken 2 is entirely installable and users can begin their assessment immediately after installation is finished. However, installation needs some bioinformatic expertise.

Moreover, two databases are used within these pipelines: one corresponding to the human genome reference, utilized by de-hosting tools, and another of microorganisms, required by taxonomic classification methods. For DRAGEN, these databases are already integrated into the tool, so the user only needs to select one of the available options. However, Kraken 2 requires the download and construction of the taxonomic database before execution. This process needs to be performed only once but requires RAM space at least equal to the database size. Furthermore, BWA and Rsubread demand indexing the human reference genome database; despite this being a one time process, it implies increasing computational resources.

### Variability in human DNA removal: influence of alignment-based de-hosting and taxonomic classification methods

**Fig. 2:**
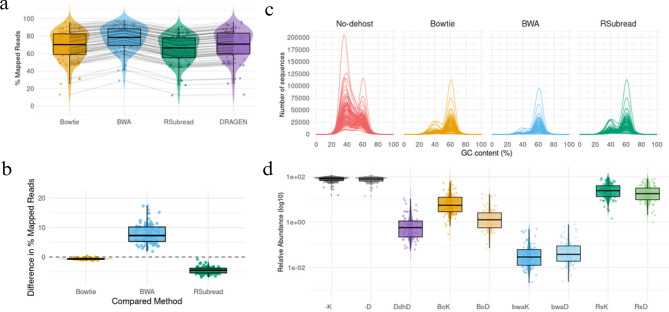
(**a**) Mapped reads to human reference for each method. (**b**) Differences in mapped reads between each method and DRAGENs in-built dehosting. (**c**) GC content by de-hosting approach. (**d**) Taxonomic classification of human-derived abundances by method

The de-hosting process refers to the identification and subtraction from the raw FASTQ sequence files (quality filtered) of the host DNA content through the use of alignment tools. As shown in Fig. 2, the de-hosting process was performed using three external alignment tools (BWA, Bowtie2, and Rsubread) and with the DRAGEN built-in de-hosting feature.

Figure 2a shows the distribution of the percentage of reads mapped to the human genome that were subtracted (de-hosted) from the trimmed sequence raw data. High amounts of reads were identified as human-derived (~70%) with significant differences between each and every pair of methodologies (Wilcoxon paired test with BH correction). Furthermore, comparing all the methods against DRAGEN (the method to compete with), Fig. 2b highlights the specific differences between all the methods against it. Notably, BWA identified a significantly higher percentage of reads mapped to the human genome (~8%); whereas Bowtie2 exhibited the smallest deviation from DRAGEN among all the evaluated methods, setting this method as the most promising in terms of de-hosting.

Analyzing the de-hosted files, we focused on the GC content, which refers to the percentage of guanine (G) and cytosine (C) bases in a DNA molecule relative to its total nucleotide composition. Fig. 2c shows the distribution of the GC content across three de-hosting techniques and no de-hosting. In the “No-dehost” panel, a prominent peak is observed around 40% GC content, which is likely to correspond to human-derived sequences, since the average GC content of the human genome is around this value [29]. A smaller secondary peak is also visible around 60% GC, suggesting the presence of bacterial sequences, mainly *Cutibacterium acnes*, one of the most abundant bacterial species in these samples and known to have a genomic GC content of around 60% [30]. In contrast, the three other panels in Fig. 2c representing samples processed with de-hosting methods show a significant reduction in the 40% GC peak, leaving the new maximum peak in sequences around 60% GC. Furthermore, we observed that samples processed with BWA yielded lower total sequence counts for both GC content peaks (~40% and ~60%) compared to RSubread and Bowtie2. For instance, BWA indicated between 9 and 48% less number of sequences than Bowtie at peak ~60% and between 19 and 83% at peak ~40%. This suggests that BWA removed sequences with GC values that were associated not only with the human genome, but also with bacteria. DRAGENs methodology was excluded from this analysis because we can not access FASTQ files generated after human DNA removal.

Despite the de-hosting pre-processing step, the taxonomic classification performed by Kraken2 and DRAGEN still identify reads as human-derived, highlighting the importance of including host sequences in the TxDB. The distribution of these relative abundances are shown in Fig. 2d for all nine evaluated pipelines. Each pipeline represents a combination of one de-hosting method (including no de-hosting) with one taxonomic identification method, following the naming scheme in Supplementary Table S1. As expected, the two strategies that avoid a previous step of alignment-based de-hosting (‘−K’ and ‘−D’) identified the greatest amount of relative abundance of *Homo sapiens*, since no reads mapped to the human reference have been removed. Yet, their abundances’ differences were significant. All comparisons between all methodologies presented significant differences between each pair of methodologies (*p* < 4.3e-15).

### Divergences in microbial identification across analytical approaches

Taxonomic identification in microbiome studies involves classifying these bacteria into hierarchical levels, which allows researchers to analyze microbial diversity, detect specific taxa associated with health or disease, and understand their contributions to the skin ecosystem.

To assess the impact of different methodologies on bacterial identification, the resulting de-hosted FASTQ files as well as no-dehosted were fed to both taxonomic identification methods, Kraken and DRAGEN.

**Fig. 3:**
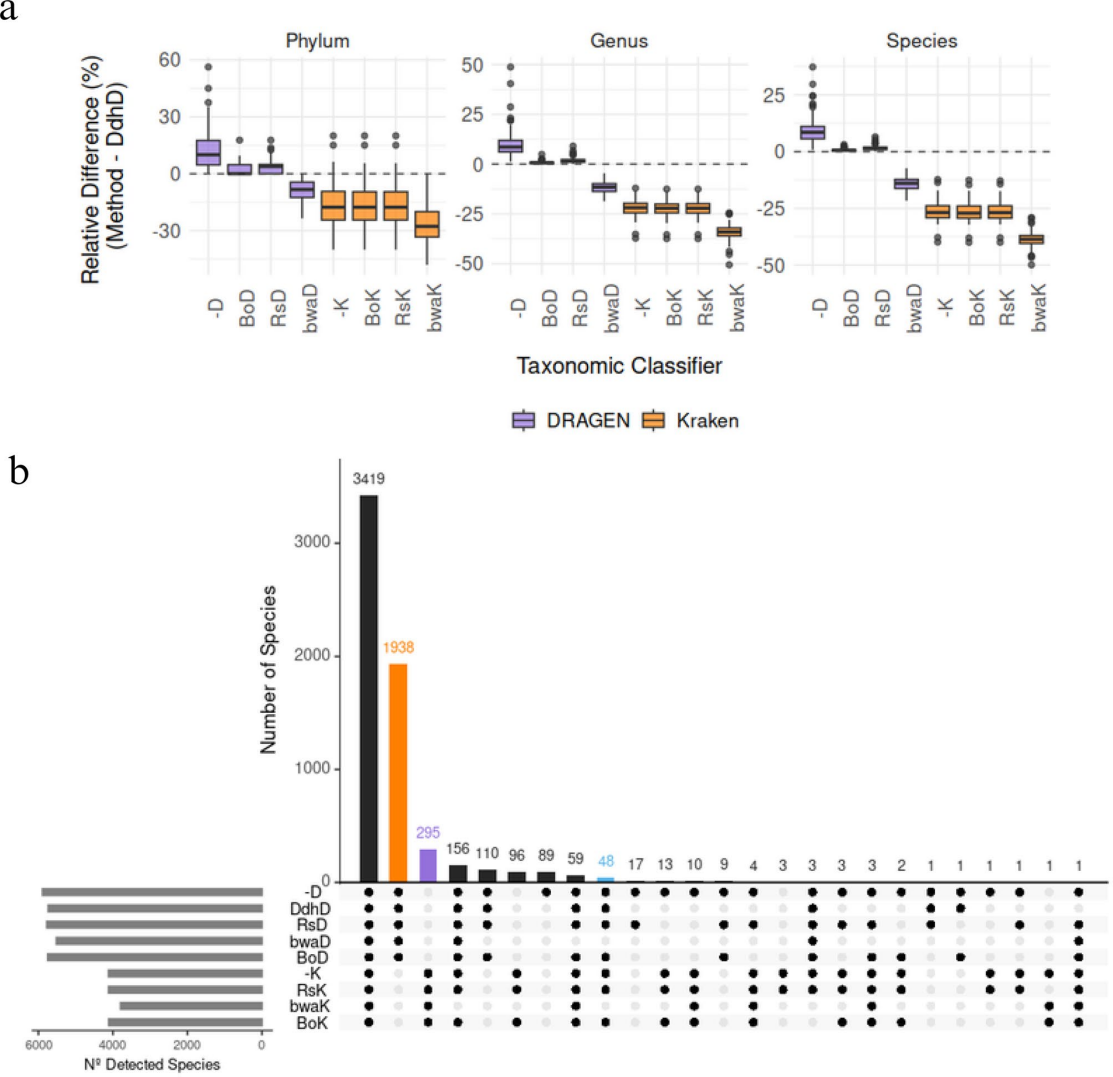
Taxonomic comparison of methodologies. (**a**) Microorganism detection differences between each method and ‘DdhD’ across taxonomic levels. (**b**) Upset plot showing shared and unique species identified by each approach

Figure 3a represents the relative differences between the number of microorganisms detected by each method against those detected by ‘DdhD’ (DRAGEN with its own de-hosting enabled), along three taxonomic levels: Phylum, Genus and Species.

Surprisingly, despite DRAGEN claiming to use the same database as Kraken, our results show that those methodologies that used DRAGEN for taxonomy classification consistently identified more microorganisms than those using Kraken. Whereas, regardless of the taxonomic classifier applied, the use of BWA systematically resulted in a lower number of detected taxa across all levels (~10% lower than Bowtie’s approaches).

The differences yielded by Bowite and Rsubread when using DRAGEN can be primarily attributed to the built-in de-hosting procedure used by the DRAGEN proprietary platform. In contrast, the discrepancies observed when comparing DRAGEN to Kraken suggest that, despite what it is claimed, the underlying taxonomic databases used by each classifier may differ.

Finally, since ‘BoD’ was the method that yielded less difference with ‘DdhD’ across all taxonomic levels, Bowtie2 emerged as the most promising and transparent alternative to the built-in DRAGEN de-hosting.

Having observed in Fig. 3a the differences in the number of species detected by each method, we proceeded to analyze Fig. 3b to explore whether the additional species identified are exclusive to a particular pipeline or shared among them. This distinction turns crucial for two main reasons,it allows evaluating whether the undetected taxa are of dermatological relevance—an aspect that could directly influence clinical decision-making and to disentangle the definition of the DRAGEN reference database as the one provided by Kraken yielding to determine whether DRAGEN has the capacity of detecting all species identified by Kraken and more, or if each method recovers a distinct set of taxa.

Fig. 3b addresses this by showing bars that represent the number of species shared by the methodologies indicated with black dots. These revealed that 3419 species were detected by all pipelines (first bar), 1938 were exclusively detected by DRAGEN pipelines (second bar), 295 exclusively by Kraken pipelines (third bar).

Among those former 1938 species, we found that 90% of them were not included in Kraken’s database (See Supplementary Material - Code Reproducibility), leading to the idea that both taxonomic databases are not equivalent, incoherent with reported information in DRAGENs documentation.

Among those latter 295 species, some are relevant to human skin. For instance, *Actinomyces meyeri* can cause actinomycosis [31]; *Betapapillomavirus 6* infects skin epithelial cells and is associated with cutaneous lesions [32]; *Bartonella schoenbuchensis* is implicated in dermatitis cases [33] and also *Propionibacterium sp. oral taxon 193,* known as *Cutibacterium modestum* (linked to acne [32]), was detected by all Kraken methodologies in at least 99% of samples.

In particular, there were 48 species detected by all pipelines except those using BWA (9th bar). For instance, *Bartonella vinsonii* can be involved in skin infections [34], *Borrelia afzelii* can cause Lyme disease [35], *Rickettsia amblyommatis* and *Rickettsia endosymbiont of Ixodes scapularis* may cause diseases related with ticks and generate cutaneous effects [36, 37].

These findings suggest that the use of BWA as de-hosting procedure may result in missing important dermatological information, meanwhile the use of Kraken-based pipeline, specially with Bowtie de-hosting step, may provide meaningful clinical dermatological information, turning the‘BoK’ metagenomic pipeline as the most robust and clinically valuable option.

### BWA de-hosting strategy significantly affect relative abundance agreement between methods

In the analysis of microbial data, the abundance estimation is a fundamental step since it provides critical insights into the taxonomic composition of a sample. Understanding these abundances allows researchers to detect patterns and identify potential associations with environmental or host-related factors, making it a key metric in comparative analyses [38, 39]. Thus, in order to evaluate if external de-hosting software may replace the built-in DRAGEN method and if the DRAGEN pipeline can be replaced by Kraken taxonomic classification, an agreement between the estimated abundances across pipelines should be conducted. In this regard, the Bland-Altman plot is proposed to visualize and analyze the differences against their mean of relative abundances assigned by each method compared to its competitor [27]. The analysis was conducted over six genera, previously proposed for the optimization of metagenomics workflows from the skin genomic mock community ATCC MSA-1005 [40]: *Acinetobacter*, *Corynebacterium*, *Cutibacterium*, *Staphylococcus*, *Streptococcus*, and *Micrococcus*. These represented between 37 and 99% of the total genera abundances (91% on average) within all our analyzed samples.

**Fig. 4:**
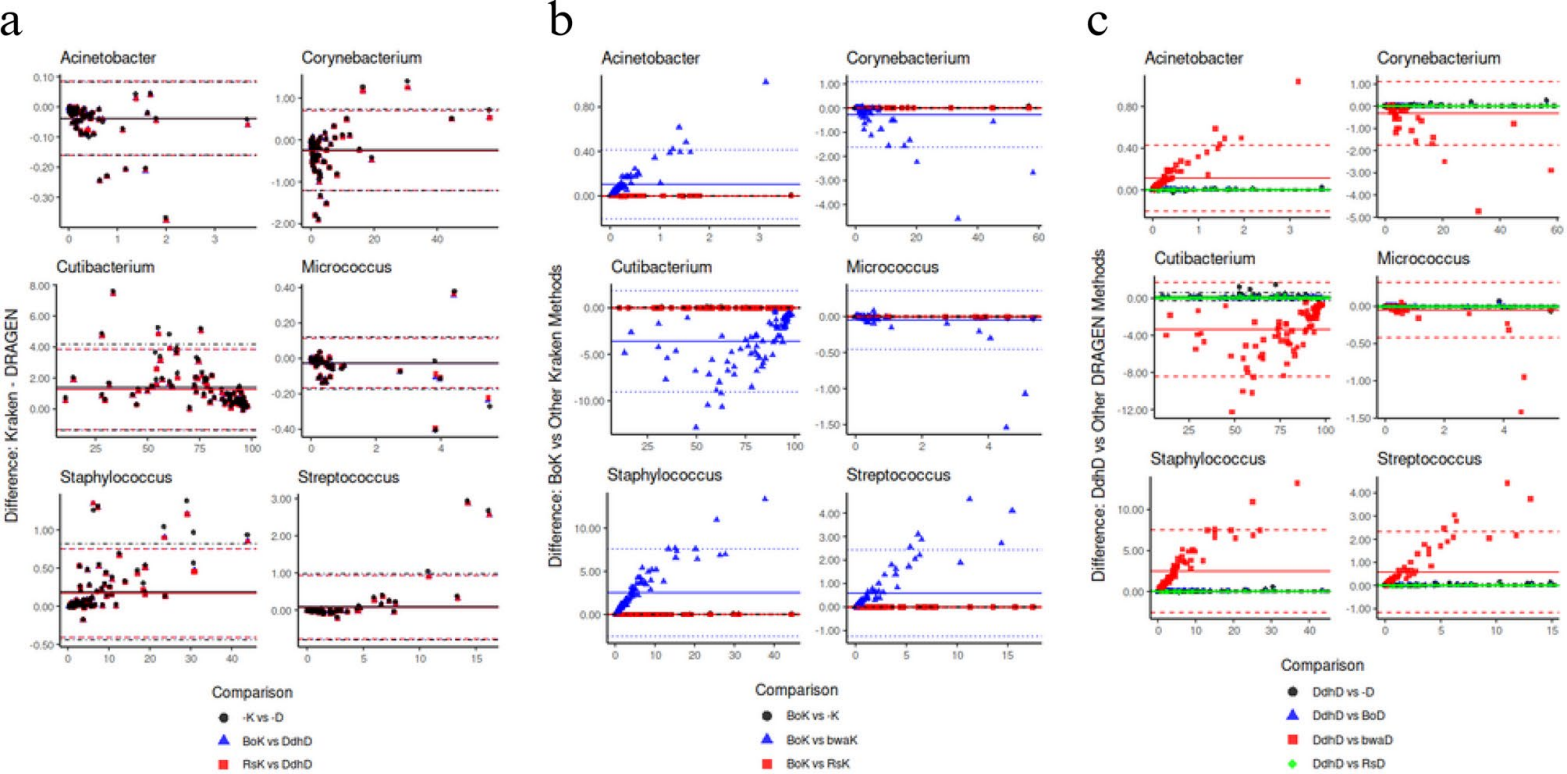
Bland-Altman plots of abundances assigned by methodology for six genera. (**a**) Alternative methods (‘BoK’ and ‘RsK’) vs. baseline method (‘DdhD’). (**b**) Bowtie2–Kraken vs. other Kraken-based methods. (**c**) DRAGEN–DRAGEN vs. other DRAGEN-based methods

To achieve a comprehensive analysis of the main impact on relative abundances estimations, three comparisons were made: ‘BoK’, established as our proposed alternative to ‘DdhD’, the method to be replaced (Fig. 4a); ‘BoK’ vs other de-hosting methods with Kraken-based for taxonomic classification (Fig. 4b) and ‘DdhD’, used as our reference, vs de-hosting methods but DRAGEN-based for taxonomic classification to evaluate de-hosting alternatives in competing pipelines (Fig. 4c).

Fig. 4a shows that the deviations of relative abundances between ‘BoK’ and ‘DdhD’, ‘RsK’ and ‘DdhD’ or ‘−K’ and ‘−D’ do not follow a consistent pattern across the six genera. Particularly ‘BoK’ and ‘RsK’ show exact same deviations, whereas ‘−K’ yields higher deviations for *Cutibacterium* and *Staphylococcus*. Therefore, ‘BoK’ and ‘RsK’ demonstrate their potential as promising pipelines in the detection of equivalent taxonomic abundances, following the results of previous analysis.

Additionally, when comparing de-hosting procedures within the same taxonomic classifier (Figs. 4b and 4c), more pronounced differences emerged, particularly when using BWA. We can observe the same pattern in both figures: BWA tends to consistently underestimate abundances for *Acinetobacter, Staphylococcus* and *Streptococcus* and overestimate Corynebacterium, *Cutibacterium* and *Micrococcus*, regardless of the taxonomic classification tool.

In contrast, comparisons between ‘BoK’ and ‘RsK’ for genera *Corynebacterium* and *Cutibacterium*, show data points distributed closer to the horizontal line at difference ≅ 0 and overlapped, representing non-significant discrepancies and suggesting ‘RsK’ as equivalent to ‘BoK’. The same thing happens with ‘DdhD’ vs ‘−D’ for *Acinetobacter*. This suggests that the absence of alignment-based de-hosting prior to taxonomic identification did not significantly alter the relative abundances assigned to this genus, assuming that DRAGENs taxonomic database contains human taxa.

### Age and sex differences in bacterial identification: how methodological variability affects clinical applications

**Fig. 5:**
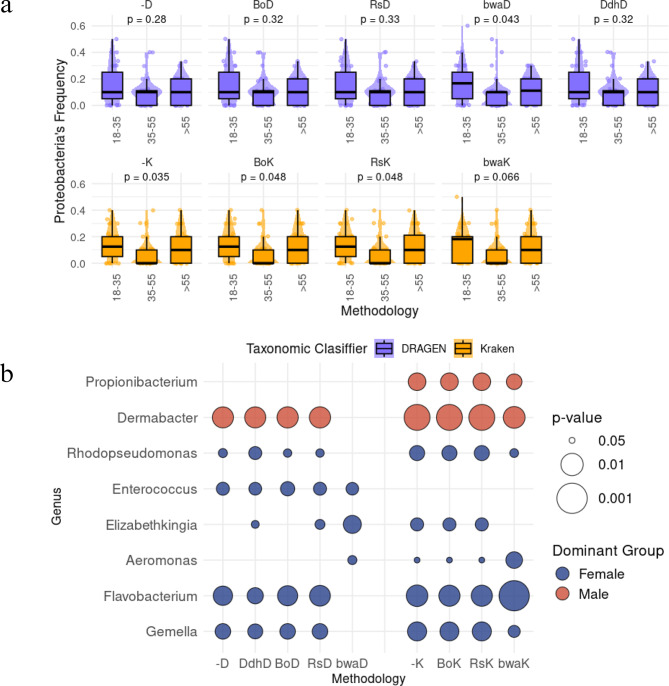
Methodological impact on taxonomic profiles. (**a**) *Proteobacteria* frequency among top 10 most abundant species by age group. (**b**) Relative abundance differences by sex across eight genera

To gain a deeper understanding of how the observed differences between processing pipelines might influence real-world applications, we turned to the metadata associated with age and sex. Nowadays, dermatologists recognize that these factors not only play a crucial role in shaping the skin microbiome but also influence the effectiveness of dermatological products [41–43]. Particularly, a common practice in dermatological studies is to analyze the most abundant bacterial taxa on the skin [44, 45], where significant shifts in microbial communities have been reported across different age groups [41, 42, 44, 46]. For instance, Luna et al. reported that *Proteobacteria*’s abundance is higher during youth, declines with age, and increases again in older individuals [47]. With the aim of reproducing this pattern, the content of *Proteobacteria* was evaluated. Fig. 5a presents the distribution of *Proteobacteria* frequencies within the top 10 most abundant species, detected by different pipelines and grouped by age range. Our findings aligned with the reported trend; however, only certain methodologies showed statistically significant differences (Kruskal-Wallis test) between age groups. Specifically, ‘bwaD’ exhibited significant differences, while all Kraken pipelines did as well—except for the one using BWA as a de-hosting method (‘bwaK’). This highlights that the use of ‘−D’, ‘BoD’, ‘RsD’, ‘DdhD’ and ‘bwaK’ may miss the significant differentiation of already published patterns, highly relevant in skin microbiome analysis.

Similarly to ageing, sex differences have been well-documented, with males and females exhibiting distinct microbial profiles at different taxonomic levels [45]. These variations are crucial when prescribing skincare products or interventions, as different microbiome profiles may require specific treatment approaches [48].

Among 1126 genera identified across all methods, 251 (22%) exhibited significant sex-related differences according to at least one method, while remaining non-significant in at least one other (Supplementary Figure S1). Of these, 37 were significant when using Kraken, whereas 31 with DRAGEN. Additionally, 19 genera showed significant sex-related differences across all methods except those using BWA. Among those 1126 genera, we identified 8 that were previously associated with sex and reported as clinically relevant. Fig. 5b illustrates their resulting significant association with sex according to different pipelines. For instance, *Propionibacterium* resulted significantly higher in males exclusively using Kraken’s pipelines, proving its reliability since they were consistent with prior findings [45]. Although Propionibacterium is now classified as *Cutibacterium*, both tools still use the names differently. DRAGEN reported several *Cutibacterium* species under this genus (C. *australiense and C. freudenreichii*), while Kraken includes taxa still labeled as Propionibacterium. This reflects database differences, complicating cross-platform comparisons for this genus.

On the other hand, *Dermabacter*, *Rhodopseudomonas, Flavobacterium and Gemella* showed differential abundances for every methodology except for ‘bwaD’, and *Elizabethkingia* only for specific methodologies [49, 50]. There were also cases such as *Aeromonas*, where all methods involving Kraken and ‘bwaD’ found significant differences between sexes, whereas the remaining methods did not. The *Gemella*, *Flavobacterium*, and *Aeromonas* genera may provide relevant information since they have been associated with dysbiosis and secondary infections [9, 51, 52].

Supplementary Figure S2 illustrates the same analyses but conducted between age groups.

These results emphasize that the use of Kraken, specifically ‘BoK’ and ‘RsK’, yields results that align with well-established microbial patterns for sex and age.

### Divergences in pathway identification with clinical importance due to de-hosting methods: BWA may mask relevant information

Identifying pathways related to the synthesis of essential compounds, such as collagen and vitamins, offers a guide for dermatologists to recommend targeted skincare products or treatments that support beneficial microbial functions [53, 54]. Therefore, analyzing the impact of de-hosting techniques in this field becomes a crucial step towards the best practice for dermatological metagenomics analysis of real-world samples.

**Fig. 6:**
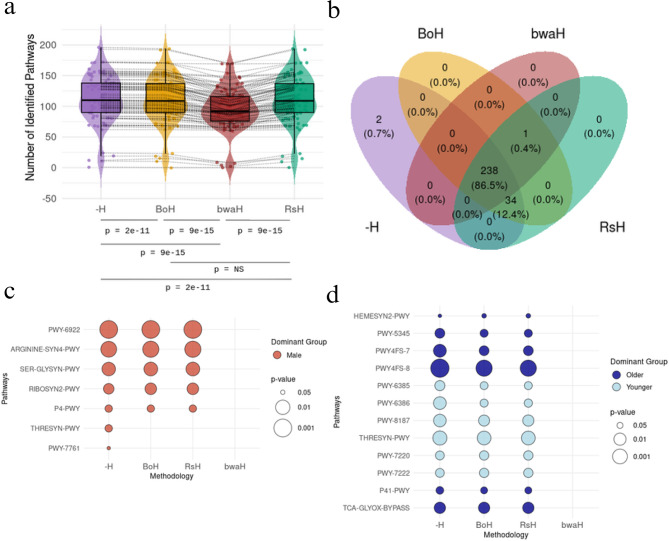
(**a**) Number of pathways per sample across by method. (**b**) Venn diagram of shared and unique pathways. (**c**) Differences in pathway abundance patterns between de-hosting methods with respect to sex and (**d**) to age

Figure 6a shows that the total number of pathways identified per sample was significantly lower when using BWA (*p* < 9e-15) and that there were no significant differences when comparing ‘BoH’ with ‘RsH’.

We also analyzed the intersection of pathways identified by each methodology for the purpose of acknowledging their dermatological importance (Fig. 6b). Two metabolic pathways, PWY-6857 and PWY-7434, were exclusively detected without de-hosting and PWY-7242 was detected by all methods except for ‘−H’. However, these pathways were identified in just one sample.

Additionally, 34 pathways were missed by BWA but detected by all the other pipelines (Supplementary Table S2). One of them was the PWY-561 pathway, described as the superpathway of the glyoxylate cycle and fatty acid degradation [55] and associated with nicotinamide adenine dinucleotide (NAD) consumption [42]. This defines it as an important therapeutic pathway since niacinamide-based skincare products support skin barrier integrity and reduce inflammation. Another example is PWY-3781, associated with oxidation and glycosylation processes, which involve clinical implications since a growth in these metabolic cascades could result in dermatological recommendations such as Vitamin C, Resveratrol or Carnosina. This pathway was found in 97,6% of the samples.

Analyzing pathway abundances in relation to aging and sex is a common approach in metagenomic studies, offering a deeper understanding of how these factors influence microbiome functionality [41, 42, 56]. We found that out of the 238 pathways identified by all methodologies, 16 (7%) showed significant differences between sexes according to at least one method, while remaining non-significant in at least one other (Supplementary Table S3). Fig. 6c illustrates seven of these pathways which were associated with clinical implications. For instance, five pathways—PWY-6922, ARGININE-SYN4-PWY, SER-GLYSYN-PWY, RIBOSYN2-PWY, and P4-PWY—showed significant association with sex in all methodologies except for BWA. While THRESYN-PWY and PWY-7761 resulted significantly higher for males only for the methodology that avoids a prior de-hosting step (‘−H’).

SER-GLY SYN-PWY pathway is associated with metabolites like glycerol (related to skin barrier function and hydration), ARGININE-SYN4-PWY, PWY-6922, THRESYN-PWY and P4-PWY are involved in L-ornithine, arginine and threonine biosynthesis respectively. These amino-acids have also been reported sex-specific concentration differences [56–58]

Moreover, RIBOSYN2-PWY pathway is related to the biosynthesis of riboflavin (vitamin B2), which is essential for energy metabolism, biosynthesis, and detoxification processes. Riboflavin deficiency is prevalent worldwide and impacts women’s health due to increased demands linked to reproductive health and hormonal fluctuations [59]. These higher demands could limit riboflavin synthesis in women compared to men. Furthermore, MetaCyc’s description of PWY-7761 also associates it with niacinamide [60], which underscores the clinical relevance of appropriate interpretation of these pathways in dermatology applications [61].

Building on the previous analysis, we examined how metabolic pathway abundances varied across age groups depending on the implemented methodology (Supplementary Table S4). Among the 238 pathways consistently identified across all methodologies, 28 (12%) displayed age-related differences that were significant in at least one method, yet not in at least one other. Notably, in 19 of these cases, the difference between age groups was significant across all methods except for BWA. For instance, HEMESYN2-PWY, PWY-5345, PWY4FS-7, and PWY4FS-8 showed significantly higher abundance in older samples when analyzed using the methodologies ‘−H’, ‘BoH’, and ‘RsH’ (Fig. 6d), but not with ‘bwaH’. This pattern is consistent with the findings reported by Chuqing Sun et al. [31], who also observed increased abundances of these pathways in older individuals. The same study identified the pathways PWY-6385, PWY-6386, and PWY-8187 as significantly more abundant in younger individuals compared to older ones. Our results presented in Figure 7b confirm these significant differences across all methodologies except ‘bwaH’.

Additionally, Chuqing Sun et al. established a categorization for microbiome-related pathways in dermatology: three ageing related categories and other three anti-aging related categories. Following this framework, THRESYN-PWY, PWY-7220, and PWY-7222 pathways were associated with anti-oxidation, reporting significantly higher abundance in younger individuals. Similarly, the same study classified P41-PWY and TCA-GLYOX-BYPASS pathways within the oxidation/glycosylation category, identifying higher abundance in older age groups. Again, our results in all methodologies except ‘bwaH’ align with these observations.

All in all, the above results emphasize that the use of BWA leads to the loss of relevant information in clinical dermatology.

## Discussion

Several studies have highlighted the need for benchmarking both de-hosting methods [62] and bacterial identification techniques to establish best practice guidelines [63]. Recently several de-hosting strategies like KneadData [64], BWA, Bowtie (aligned-based) and KrakenUniq, KMCP [65], STAT (k-mer based) were benchmarked [12, 17]. However, KneadData is a wrapper method over the Bowtie alignment tool, whereas KrakenUniq, STAT and KMCP are a taxonomy classifiers (k-mer based) where the de-hosting procedure depends on the presence of the target host taxa in the reference taxa database.

Here the main objective was set on the need for sample processing access democratization for dermatological applications, overcoming the limitations of commonly used software platforms like the DRAGEN metagenomic pipeline from the Illumina BaseSpace platform, disentangling the importance of understanding the impact of alignment-based de-hosting methods attached to k-mer based taxonomic classification for clinical dermatological applications.

In this sense, it is demonstrated that de-hosting procedure is a must as well as the inclusion of the host taxa in the reference taxa database for taxonomic classification and functional analysis [16]. In addition, the main effects of using different technical approaches, not only in terms of the bioinformatics skills required but also in the results obtained at taxonomic and functional levels was revealed.

Our results emphasize the importance of including human taxa on k-mer based taxonomic classification tools, for two main reasons: i) all the alignment-based de-hosting methods shown here can not completely remove human reads from sequenced samples and ii) bacterial assembled genomes may be contaminated by human DNA as stated in Breitwieser et al. and Gihawi et al. In this regards, we strongly recommend a) to perform aligned-based host DNA removal (avoid using BWA method), despite clinically relevant taxonomic abundances and metabolic pathways were, in the present study, consistent across analyses performed with and without Bowtie, Rsubread or Built-In DRAGEN de-hosting tools, and b) include the host/human taxa in the k-mer based taxonomic classification tool reference taxa database. In addition, de-hosting may reduce storage demands and ensure sample privacy [66].

Regarding microorganism identification and metabolic pathway analysis, our findings show that the pipelines employed were robust across different de-hosting techniques, with the exception of BWA. While Bowtie2 remains the most widely used option, Rsubread offers a practical alternative for those seeking to avoid additional installations. In contrast, BWA consistently produced divergent results across all comparisons, showing significant discrepancies, identifying fewer taxa and pathways, and failing to capture well-established microbiome patterns. Such omissions could lead to misinterpretation of results, potentially compromising the accuracy of clinical insights and undermining the reliability of skin-related microbiome research.

Another interesting fact uncovered is that, despite Illumina’s DRAGEN documentation stating that it relies on the same algorithms and databases as Kraken, our results revealed notable differences between these methodologies regarding microorganisms identified and age-related established patterns in key bacterial groups. This raises concerns about methodological and database consistency.

Rather than simply confirming tool-dependent variability, this study reveals how specific combinations affect clinically relevant outputs for dermatological applications. Our results suggest the inclusion of Bowtie2 or Rsubread for de-hosting and Kraken 2 for taxonomic classification, to make sure the pipeline and databases are customizable and its results will be aligned with previously established microbiome patterns.

## Conclusions

Our findings show that for dermatologists seeking a general overview of the most abundant skin species, the DRAGEN metagenomic pipeline with de-hosting enabled provided by Illumina BaseSpace offers a practical solution, requiring minimal bioinformatics expertise. But to gain control on the bioinformatic pipeline, analytical versatility, data integration and automation for further downstream analysis the Kraken2 attached with Bowtie 2 or Rsubread (easier installation on the R language platform) de-hosting procedures is strongly recommended. In this regard, Kraken 2 with Bowtie/Rsubread emerges as a more precise and customizable option. Furthermore, when the goal is to identify rare or less abundant species that may play a role in skin health, despite DRAGEN proves effective in detecting a broader variety of bacteria, their lack of access to the underlying database and algorithms raised concerns about transparency, as it became unclear whether the databases and algorithms truly match those of Kraken 2.

Consequently, our results underscored the critical importance of selecting the appropriate methodology, as an inadequate choice can obscure key biological patterns and hinder accurate clinical analysis. These discrepancies emphasize that using the wrong approach may miss biologically and clinically relevant signatures, ultimately compromising the reliability of microbiome-based dermatological assessments and potentially leading to misinterpretations.

Rather than endorsing a single best practice, this study serves as a guide to help professionals—clinicians, dermatologists, and researchers—in selecting the most appropriate methodology based on their specific biological question and available resources. By understanding these methodological differences, professionals can make informed decisions that enhance the characterization of the skin microbiome, improve diagnostic and therapeutic applications, and ultimately contribute to advancing dermatological research and patient care.

This study not only illustrates the technical differences between these tools but also emphasizes their clinical relevance, particularly in the context of personalized dermatological treatments. As microbiome data becomes increasingly integrated into dermatology, understanding how these tools affect species detection and subsequent interpretations is key to advancing clinical practices and enhancing patient outcomes.

## Electronic supplementary material

Below is the link to the electronic supplementary material.

Supplementary material 1

## Data Availability

The raw data that support the findings of this study are not publicly available due to confidentiality agreements and ethical restrictions concerning sensitive participant information, but are available from the corresponding author on reasonable request. However, a GitHub repository containing the code and processed data necessary to reproduce the analyses and figures presented in this manuscript is available at:https://github.com/DanielaOrschanski/PipelinesMetagenomics.

## Abbreviations

BWA: Burrows-Wheeler Aligner
DRAGEN: Illumina DRAGEN’s Metagenomic Pipeline
QC: Quality Control
HUMAnN: HUMAnN 3.0
Bowtie: Bowtie 2
Kraken: Kraken 2
BH: Benjamini-Hochberg
