# Supplementary material for: Dermatological implications of alignment-based de-hosting and bioinformatics pipelines on shotgun microbiome analysis
Source: J Transl Med. 2025 Nov 13;23:1276. doi: 10.1186/s12967-025-07246-z (PMC12613601; doi:10.1186/s12967-025-07246-z)
Supplement: Supplementary file 1 — Supplementary material 1 [file 12967_2025_7246_MOESM1_ESM.docx]

# Supplementary Material

**Table S1:** Combination of Alignment-Based De-Hosting Methods with Taxonomy and Pathway Identification Methods. Four pipelines for Kraken 2 ( -K: un-dehosted, BoK: de-hosted by Bowtie 2, bwaK: de-hosted by BWA and RsK: de-hosted by Rsubread R package), five for DRAGEN (-D, BoD, bwaD, RsD and DdhD: de-hosted by proprietary DRAGEN de-hosting procedure) and four for HUMAnN (-H: un-dehosted, BoH, bwaH, RsH).

| **De-hosting Method** | **Downstream analysis** | **Taxonomy Identification Method** | **Resulted Methodology** |
| --- | --- | --- | --- |
| None | Taxa | Kraken 2 | -K |
|  | Taxa | DRAGEN | -D |
|  | Pathway | HUMAnN | -H |
| Bowtie 2 | Taxa | Kraken 2 | BoK |
|  | Taxa | DRAGEN | BoD |
|  | Pathway | HUMAnN | BoH |
| BWA | Taxa | Kraken 2 | bwaK |
|  | Taxa | DRAGEN | bwaD |
|  | Pathway | HUMAnN | bwaH |
| Rsubread | Taxa | Kraken 2 | RsK |
|  | Taxa | DRAGEN | RsD |
|  | Pathway | HUMAnN | RsH |
| DRAGEN’s de-hosting feature enabled | Taxa | DRAGEN | DdhD |


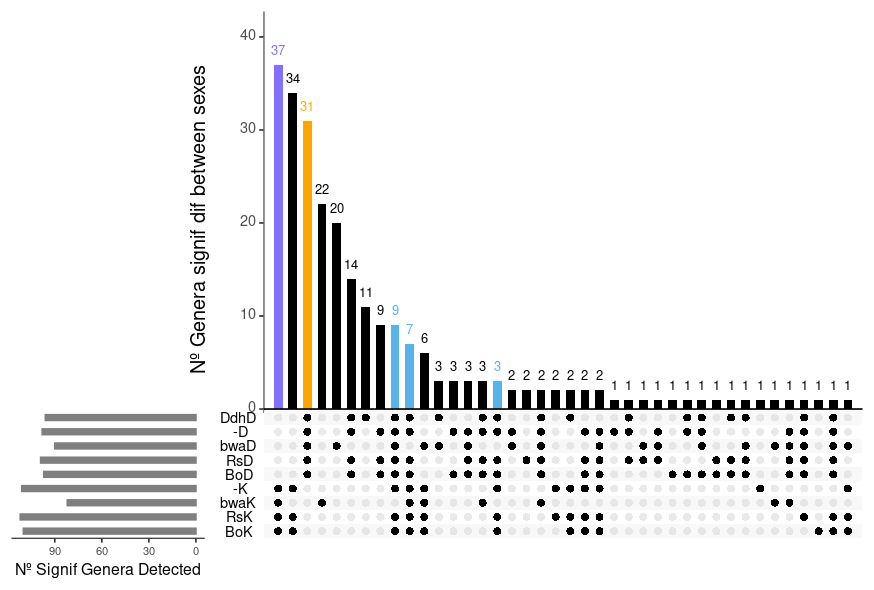


**Figure S1:** Upset plot representing which genera exhibited significant sex-related differences according to each method.


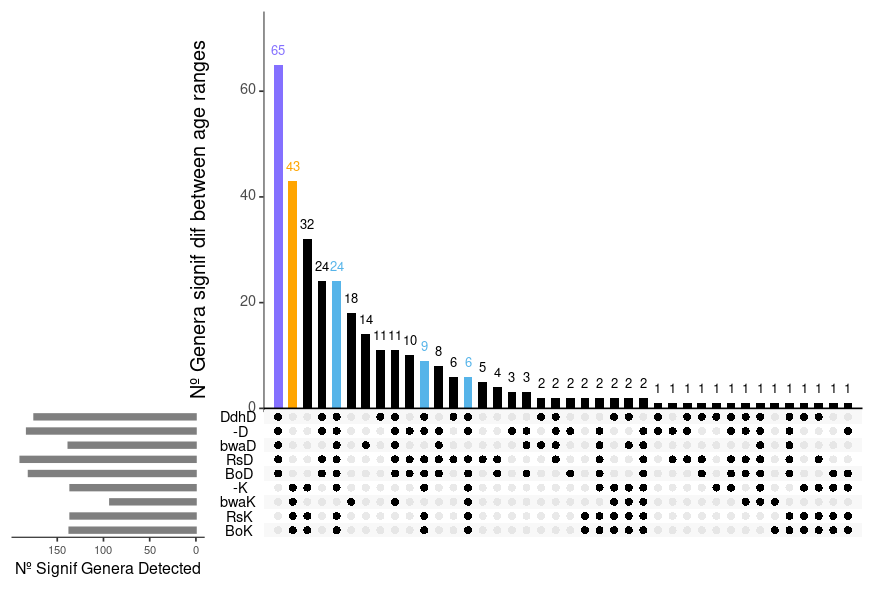


**Figure S2:** Upset plot representing which genera are found to be statistically different between age groups depending on the methodology applied.

**Table S2:** Description of pathways which were detected by all methodologies except for BWA. “% Samples” refers to the percentage of samples in which each pathway was detected.

| **Pathway** | **Class** | **Description** | **% Samples** | | | **Mean Abundance** | | |
| --- | --- | --- | --- | --- | --- | --- | --- | --- |
|  |  |  | **-H** | **BoH** | **RsH** | **-H** | **BoH** | **RsH** |
| PWY-3781 | Energy-Metabolism | aerobic respiration I (cytochrome c) | 96.39 | 1.2 | 1.2 | 3.85 | 0.03 | 0.03 |
| FOLSYN-PWY | Cofactor-Biosynthesis // Super-Pathways | superpathway of tetrahydrofolate biosynthesis and salvage | 7.23 | 7.23 | 7.23 | 0.18 | 0.18 | 0.18 |
| PWY-6612 | Cofactor-Biosynthesis // Super-Pathways | superpathway of tetrahydrofolate biosynthesis | 7.23 | 7.23 | 7.23 | 0.17 | 0.17 | 0.17 |
| PWY-6606 | NUCLEO-DEG | guanosine nucleotides degradation II | 3.61 | 3.61 | 3.61 | 0.08 | 0.08 | 0.08 |
| PWY-7184 | Nucleotide-Biosynthesis // Metabolic-Clusters | pyrimidine deoxyribonucleotides de novo biosynthesis I | 3.61 | 3.61 | 3.61 | 0.1 | 0.1 | 0.1 |
| PWY-7210 | Nucleotide-Biosynthesis // Metabolic-Clusters | pyrimidine deoxyribonucleotides biosynthesis from CTP | 3.61 | 3.61 | 3.61 | 0.1 | 0.1 | 0.1 |
| PWY-922 | SECONDARY-METABOLITE-BIOSYNTHESIS | mevalonate pathway I (eukaryotes and bacteria) | 3.61 | 3.61 | 3.61 | 0.08 | 0.08 | 0.08 |
| P108-PWY | Energy-Metabolism | pyruvate fermentation to propanoate I | 2.41 | 1.2 | 1.2 | 0.06 | 0.03 | 0.03 |
| PWY-6549 | Transport-Pathways | L-glutamine biosynthesis III | 2.41 | 2.41 | 2.41 | 0.06 | 0.06 | 0.06 |
| PWY-7117 | Energy-Metabolism | C4 photosynthetic carbon assimilation cycle. PEPCK type | 2.41 | 2.41 | 2.41 | 0.05 | 0.05 | 0.05 |
| PWY-7185 | NUCLEO-DEG | UTP and CTP dephosphorylation I | 2.41 | 2.41 | 2.41 | 0.06 | 0.06 | 0.06 |
| KETOGLUCONMET-PWY | Super-Pathways // CARBOXYLATES-DEG | ketogluconate metabolism | 1.2 | 1.2 | 1.2 | 0.03 | 0.03 | 0.03 |
| METHGLYUT-PWY | Super-Pathways // Aldehyde-Degradation | superpathway of methylglyoxal degradation | 1.2 | 1.2 | 1.2 | 0.03 | 0.03 | 0.03 |
| P122-PWY | Energy-Metabolism | heterolactic fermentation | 1.2 | 1.2 | 1.2 | 0.03 | 0.03 | 0.03 |
| P124-PWY | Energy-Metabolism // Carbohydrates-Degradation | Bifidobacterium shunt | 1.2 | 1.2 | 1.2 | 0.03 | 0.03 | 0.03 |
| P164-PWY | Energy-Metabolism // NUCLEO-DEG | purine nucleobases degradation I (anaerobic) | 1.2 | 1.2 | 1.2 | 0.03 | 0.03 | 0.03 |
| PHOTOALL-PWY | Energy-Metabolism // Super-Pathways | oxygenic photosynthesis | 1.2 | 1.2 | 1.2 | 0.03 | 0.03 | 0.03 |
| PWY-5130 | Super-Pathways // CARBOXYLATES-DEG | 2-oxobutanoate degradation I | 1.2 | 1.2 | 1.2 | 0.03 | 0.03 | 0.03 |
| PWY-5367 | Lipid-Biosynthesis | petroselinate biosynthesis | 1.2 | 1.2 | 1.2 | 0.03 | 0.03 | 0.03 |
| PWY-561 | Energy-Metabolism // Super-Pathways | superpathway of glyoxylate cycle and fatty acid degradation | 1.2 | 1.2 | 1.2 | 0.03 | 0.03 | 0.03 |
| PWY-5838 | Cofactor-Biosynthesis // Super-Pathways | superpathway of menaquinol-8 biosynthesis I | 1.2 | 1.2 | 1.2 | 0.03 | 0.03 | 0.03 |
| PWY-5861 | Cofactor-Biosynthesis // Super-Pathways | superpathway of demethylmenaquinol-8 biosynthesis I | 1.2 | 1.2 | 1.2 | 0.03 | 0.03 | 0.03 |
| PWY-5873 | Cofactor-Biosynthesis | ubiquinol-7 biosynthesis (late decarboxylation) | 1.2 | 1.2 | 1.2 | 0.03 | 0.03 | 0.03 |
| PWY-5971 | Lipid-Biosynthesis | palmitate biosynthesis (type II fatty acid synthase) | 1.2 | 1.2 | 1.2 | 0.03 | 0.03 | 0.03 |
| PWY-6284 | Lipid-Biosynthesis // Super-Pathways | superpathway of unsaturated fatty acids biosynthesis (E. coli) | 1.2 | 1.2 | 1.2 | 0.03 | 0.03 | 0.03 |
| PWY-6305 | Polyamine-Biosynthesis // Super-Pathways | superpathway of putrescine biosynthesis | 1.2 | 1.2 | 1.2 | 0.03 | 0.03 | 0.03 |
| PWY-6545 | Nucleotide-Biosynthesis // Metabolic-Clusters | pyrimidine deoxyribonucleotides de novo biosynthesis III | 1.2 | 1.2 | 1.2 | 0.03 | 0.03 | 0.03 |
| PWY-6876 | Energy-Metabolism | isopropanol biosynthesis (engineered) | 1.2 | 1.2 | 1.2 | 0.03 | 0.03 | 0.03 |
| PWY-7039 | Lipid-Biosynthesis | phosphatidate metabolism. as a signaling molecule | 1.2 | 1.2 | 1.2 | 0.03 | 0.03 | 0.03 |
| PWY-7211 | Nucleotide-Biosynthesis // Super-Pathways | superpathway of pyrimidine deoxyribonucleotides de novo biosynthesis | 1.2 | 1.2 | 1.2 | 0.03 | 0.03 | 0.03 |
| PWY66-367 | Energy-Metabolism | ketogenesis | 1.2 | 1.2 | 1.2 | 0.03 | 0.03 | 0.03 |
| PWY66-391 | Fatty-Acid-and-Lipid-Degradation | fatty acid &beta;-oxidation VI (mammalian peroxisome) | 1.2 | 1.2 | 1.2 | 0.03 | 0.03 | 0.03 |
| SALVADEHYPOX-PWY | NUCLEO-DEG | adenosine nucleotides degradation II | 1.2 | 1.2 | 1.2 | 0.03 | 0.03 | 0.03 |
| THISYN-PWY | Cofactor-Biosynthesis // Super-Pathways | superpathway of thiamine diphosphate biosynthesis I | 1.2 | 1.2 | 1.2 | 0.03 | 0.03 | 0.03 |

**Table S3:** Pathways that show significant differences between sexes when analyzed with one methodology but not with others. Descriptions and classification are based on MetaCyc.

| **Pathway** | **p-values** | | | | **Class** | **Description** |
| --- | --- | --- | --- | --- | --- | --- |
|  | **-H** | **BoH** | **bwaH** | **RsH** |  |  |
| ARGININE-SYN4-PWY | 0.007 | 0.006 | 0.054 | 0.006 | Amino-Acid-Biosynthesis | L-ornithine biosynthesis II |
| RIBOSYN2-PWY | 0.023 | 0.019 | 0.263 | 0.019 | Cofactor-Biosynthesis | Flavin biosynthesis I (bacteria and plants) |
| PWY-6922 | 0.003 | 0.003 | 0.051 | 0.003 | Amino-Acid-Biosynthesis | L-N-delta-acetylornithine biosynthesis |
| SER-GLYSYN-PWY | 0.012 | 0.012 | 0.114 | 0.012 | Amino-Acid-Biosynthesis // Super-Pathways | Superpathway of L-serine and glycine biosynthesis I |
| P4-PWY | 0.036 | 0.036 | 0.311 | 0.036 | Amino-Acid-Biosynthesis // Super-Pathways | Superpathway of L-lysine, L-threonine and L-methionine biosynthesis I |
| THRESYN-PWY | 0.036 | 0.129 | 0.12 | 0.131 | Amino-Acid-Biosynthesis // Super-Pathways | Superpathway of L-threonine biosynthesis |
| PWY-7761 | 0.043 | 0.051 | 0.079 | 0.053 | Cofactor-Biosynthesis | NAD salvage pathway II (PNC IV cycle) |
| PWY66-389 | 0.063 | 0.104 | 0.031 | 0.104 | Alcohol-Degradation | Phytol degradation |
| PWY-7198 | 0.009 | 0.01 | 0.125 | 0.01 | Nucleotide-Biosynthesis // Metabolic-Clusters | Pyrimidine deoxyribonucleotides de novo biosynthesis IV |
| FERMENTATION-PWY | 0.054 | 0.054 | 0.008 | 0.054 | Energy-Metabolism // Super-Pathways | Mixed acid fermentation |
| PWY-101 | 0.028 | 0.028 | 0.175 | 0.028 | Energy-Metabolism | Photosynthesis light reactions |
| PWY-4984 | 0.027 | 0.027 | 0.056 | 0.027 | Noncarbon-Nutrients | Urea cycle |
| PWY-5265 | 0.463 | 0.48 | 0.036 | 0.48 | Cell-Structure-Biosynthesis // Super-Pathways | Peptidoglycan biosynthesis II (staphylococci) |
| PWY-5723 | 0.035 | 0.035 | 0.094 | 0.035 | Energy-Metabolism | Rubisco shunt |
| PWY-6527 | 0.008 | 0.008 | 0.094 | 0.008 | Carbohydrates-Degradation | Stachyose degradation |
| TCA | 0.155 | 0.155 | 0.015 | 0.155 | Energy-Metabolism | TCA cycle I (prokaryotic) |

**Table S4:** Pathways that show significant differences between age groups when analyzed with one methodology but not with others. Descriptions and classification are based on MetaCyc.

| **Pathway** | **P-values** | | | | **Class** | **Description** |
| --- | --- | --- | --- | --- | --- | --- |
|  | **BoH** | **-H** | **RsH** | **bwaH** |  |  |
| HEMESYN2-PWY | 0.048 | 0.049 | 0.048 | 0.434 | Cofactor-Biosynthesis // Tetrapyrrole-Biosynthesis | heme b biosynthesis II (oxygen-independent) |
| PWY-5345 | 0.032 | 0.021 | 0.032 | 0.224 | Amino-Acid-Biosynthesis // Super-Pathways | superpathway of L-methionine biosynthesis (by sulfhydrylation) |
| PWY4FS-7 | 0.022 | 0.008 | 0.022 | 0.053 | Lipid-Biosynthesis // Super-Pathways | phosphatidylglycerol biosynthesis I (plastidic) |
| PWY4FS-8 | 0.002 | 0.001 | 0.002 | 0.053 | Lipid-Biosynthesis // Super-Pathways | phosphatidylglycerol biosynthesis II (non-plastidic) |
| THRESYN-PWY | 0.006 | 0.005 | 0.005 | 0.098 | Amino-Acid-Biosynthesis // Super-Pathways | superpathway of L-threonine biosynthesis |
| PWY-7220 | 0.024 | 0.027 | 0.024 | 0.102 | Nucleotide-Biosynthesis | adenosine deoxyribonucleotides de novo biosynthesis II |
| PWY-7222 | 0.024 | 0.027 | 0.024 | 0.102 | Nucleotide-Biosynthesis | guanosine deoxyribonucleotides de novo biosynthesis II |
| P41-PWY | 0.038 | 0.034 | 0.038 | 0.059 | Energy-Metabolism // Super-Pathways | pyruvate fermentation to acetate and (S)-lactate I |
| TCA-GLYOX-BYPASS | 0.014 | 0.014 | 0.014 | 0.355 | Super-Pathways | superpathway of glyoxylate bypass and TCA |
| HEME-BIOSYNTHESIS-II-1 | 0.151 | 0.151 | 0.151 | 0.041 | Cofactor-Biosynthesis // Tetrapyrrole-Biosynthesis | heme b biosynthesis V (aerobic) |
| NAGLIPASYN-PWY | 0.271 | 0.271 | 0.271 | 0.014 | Glycan-Pathways // Lipid-Biosynthesis // Cell-Structure-Biosynthesis | lipid IVA biosynthesis (E. coli) |
| P105-PWY | 0.024 | 0.024 | 0.024 | 0.079 | Energy-Metabolism | TCA cycle IV (2-oxoglutarate decarboxylase) |
| P185-PWY | 0.049 | 0.049 | 0.049 | 0.148 | C1-COMPOUNDS | formaldehyde assimilation III (dihydroxyacetone cycle) |
| PANTO-PWY | 0.048 | 0.037 | 0.048 | 0.132 | Cofactor-Biosynthesis | phosphopantothenate biosynthesis I |
| PHOSLIPSYN-PWY | 0.041 | 0.014 | 0.041 | 0.355 | Lipid-Biosynthesis // Super-Pathways | superpathway of phospholipid biosynthesis I (bacteria) |
| PWY-5005 | 0.055 | 0.052 | 0.054 | 0.008 | Cofactor-Biosynthesis // Super-Pathways | biotin biosynthesis II |
| PWY-5030 | 0.021 | 0.061 | 0.021 | 0.123 | Amino-Acid-Degradation |  |
| PWY-6121 | 0.09 | 0.083 | 0.087 | 0.046 | Nucleotide-Biosynthesis | 5-aminoimidazole ribonucleotide biosynthesis I |
| PWY-6385 | 0.033 | 0.02 | 0.032 | 0.066 | Cell-Structure-Biosynthesis // Super-Pathways | peptidoglycan biosynthesis III (mycobacteria) |
| PWY-6386 | 0.032 | 0.008 | 0.031 | 0.053 | Cell-Structure-Biosynthesis | UDP-N-acetylmuramoyl-pentapeptide biosynthesis II (lysine-containing) |
| PWY-702 | 0.0004 | 0.0004 | 0.0004 | 0.07 | Amino-Acid-Biosynthesis | L-methionine biosynthesis II |
| PWY-7282 | 0.381 | 0.353 | 0.381 | 0.042 | Cofactor-Biosynthesis | 4-amino-2-methyl-5-diphosphomethylpyrimidine biosynthesis II |
| PWY-7761 | 0.06 | 0.072 | 0.06 | 0.044 | Cofactor-Biosynthesis | NAD salvage pathway II (PNC IV cycle) |
| PWY-8073 | 0.271 | 0.271 | 0.271 | 0.014 | Glycan-Pathways // Lipid-Biosynthesis // Cell-Structure-Biosynthesis | lipid IVA biosynthesis (P. putida) |
| PWY-8187 | 0.015 | 0.009 | 0.014 | 0.072 | Energy-Metabolism // Amino-Acid-Degradation | L-arginine degradation XIII (reductive Stickland reaction) |
| PWY1ZNC-1 | 0.039 | 0.031 | 0.039 | 0.186 | Noncarbon-Nutrients | assimilatory sulfate reduction IV |
| RIBOSYN2-PWY | 0.225 | 0.104 | 0.225 | 0.013 | Cofactor-Biosynthesis | flavin biosynthesis I (bacteria and plants) |
| TCA | 0.032 | 0.032 | 0.032 | 0.099 | Energy-Metabolism | TCA cycle I (prokaryotic) |

**Code Reproducibility**

GitHub repository containing the code and processed data necessary to reproduce the analyses and figures presented in manuscript. In this repository you will find:

- Execution scripts:
  1. DeHosting.R: Contains all the coding necessary for applying any studied de-hosting method (Bowtie2, Rsubread and BWA)
  2. RunDRAGEN-P.R
  3. RunKRAKEN-P.R
  4. RunHuman-P.R
- Scripts for reproducibility of figures:

1. Figure2.R: makes use of Fig2a-MappedReads.xlsx, Fig2c-GC.xlsx and Fig2d-HomoSapiens.xlsx.
2. Figure3.R: makes use of Fig3a-MicroorgPerLevel.xlsx, Fig3-All_Taxa_Kraken.xlsx and Fig3-data_list.RData.
3. Figure4.R: makes use of Fig4-RA6genera.xlsx.
4. Figure5.R: makes use of Fig5-DFProteobacteria.xlsx, Fig5-Genera_DifSign_Age.xlsx, Fig5-Genera_DifSign_Sex.xlsx
5. Figure6.R: makes use of Fig6a-NumberPathways.xlsx, Fig6b-DataList-Pathways.RData, Fig6c-Pathways-Sex.xlsx, Fig6d-Pathways-Age.xlsx, TableS2, TableS3, TableS4.
